# Our Friends

**Published:** 1873-03

**Authors:** 


The following named gentlemen paid for the *Companion* last year (1872), but had no receipts forwarded them. If any others have paid and have not been properly receipted, they shall be attended to promptly by informing the editors:

*Georgia.*—Dr. J. P. Clopton, $2 00; Dr. C. D. Smith, 2 00; Dr. R. H. Hightower, 2 00; Dr. C. H. Smith, 2 00; Dr. H. M. Talley, 2 00; Dr. N. Gillis, 2 00; Dr. P. H. Wright, 2 00.

*Virginia.*—Dr. W. J. Moore, 2 00; Dr. Wm. White, 2 00; Dr. R. Thompson, 2 00.

*Alabama.*—Dr. S. L. Bonner, 2 00; C. N. Candler, 2 00.

*Mississippi.*—Dr. R. F. Mathews, 2 00; Dr. I. Collins, 2 00.

*Louisiana.*—Dr. John Jones, 2 00.

*North Carolina.*—Dr. C. N. Candler, 2 00.

*Kentucky.*—Dr. H. C. Miller, 2 00.

*Our Friends*

Are respectfully asked to let us know of any mistakes that may occur in their names, post-offices, etc. We will correct all mistakes with pleasure.

